# Supplementary material for: Endemicity and prevalence of multipartite viruses under heterogeneous between-host transmission
Source: PLoS Comput Biol. 2019 Mar 18;15(3):e1006876. doi: 10.1371/journal.pcbi.1006876 (PMC6438571; doi:10.1371/journal.pcbi.1006876)
Supplement: S1 Text — We provide a thorough explanation of the analytical findings. We start from only one segmented variant (v = 1) in Section 1, then generic v (Section 2), and subsequently incorporate contact heterogeneity (Section 3). In Section 4 we include differential transmissibility of segmented variants. In Section 5 we analytically derive the total prevalence of the wild-type form. In Section 6, we provide a numerical validation of the critical surfaces in the phase space. In Section 7, we derive the critical surfaces when accounting for limited viral production by host cells. Finally, in Section 8 we consider simple corrections to our model accounting for nonhomogeneous recovery rates, and nonindependent transmission probabilities. (PDF) [file pcbi.1006876.s001.pdf]

S1 Text: Supporting Information for

Endemicity and prevalence of multipartite viruses under heterogeneous between-host transmission

Eugenio Valdano[1], Susanna Manrubia[2,3], Sergio Gómez[1], and Alex Arenas[1]

[1]Departament d'Enginyeria Informàtica i Matemàtiques, Tarragona, Spain.
[2]National Centre for Biotechnology (CSIC), Madrid, Spain.
[3]Grupo Interdisciplinar de Sistemas Complejos (GISC), Madrid, Spain.

The notation used here is the same as the one introduced in the main text. Equations and figures of the main text are referenced with their number there, e.g., Fig. 1 and Eq. (2). Equations and figures on this Supporting Information have a "S." before their number, e.g., Fig. S.1 and Eq. (S.2).

We report here the equation describing the spread of the disease, corresponding to Eq. (1) of the main text:

$$\dot{x}^k_\nu = -\mu x^k_\nu + \frac{k}{\langle k\rangle}\sum_\beta\left(\sum_h h p_\gamma(h) x^h_\beta\right)\left[\Gamma_{\nu\beta}\left(1-\sum_\gamma x^k_\gamma\right)+\sum_\gamma \Lambda_{\nu\beta\gamma}x^k_\gamma\right]. \tag{S.1}$$

The terms in this equation are described in the main text. We remind that the Latin indices run on the degree, and Greek indices run over the $2^v + 1$ compartments, that are ordered by increasing number of viral species they contain. Let us define as ϕ the function that counts such number, so that $\phi(\ulcorner\mathbf{wt}\lrcorner) = 1$ (just wt), $\phi(\ulcorner\mathbf{1}\lrcorner) = 2$ (wt plus one variant), $\phi(\ulcorner\mathbf{seg}\lrcorner) = v$ (all the variants), $\phi(\ulcorner\mathbf{all}\lrcorner) = v + 1$, and so on. The ordering is such that $\alpha > \beta \Rightarrow \phi(\ulcorner\alpha\lrcorner) \geq \phi(\ulcorner\beta\lrcorner)$.

S.1 Model $v = 1$

Here we consider $v = 1$, homogeneous contacts, and no differential degradation. Let x_1, x_2 be the prevalence of $\ulcorner\mathbf{wt}\lrcorner$, $\ulcorner\mathbf{all}\lrcorner$, respectively. Equation (S.1) reduces to

$$\begin{cases} \dot{x}_1 = -\mu x_1 + \lambda(1 - x_1 - x_2)x_1 + \lambda(1-\lambda)(1 - x_1 - x_2)x_2 - \lambda x_1 x_2; \\ \dot{x}_2 = -\mu x_2 + \lambda^2(1 - x_1 - x_2)x_2 + \lambda x_1 x_2. \end{cases} \tag{S.2}$$

As explained in the main text, the equation of the total prevalence $z = x_1 + x_2$ decouples (see also Sect. S.5). It is thus convenient to consider the system in (z, x_2):

$$\begin{cases} \dot{z} = \lambda(1-z)z - \mu z \\ \dot{x}_2 = \lambda^2(1-z)x_2 + \lambda(z - x_2)x_2 - \mu x_2. \end{cases} \tag{S.3}$$

As the equation for z decouples from x_2, the Jacobian is lower triangular:

$$J = \begin{pmatrix} -\mu + \lambda\,(1-2z) & 0 \\ \lambda(1-\lambda)x_2 & \lambda^2(1-z) + \lambda(z-2x_2) - \mu \end{pmatrix}. \tag{S.4}$$

The spectrum of J is thus given by its diagonal elements. In order to get T_1, i.e., the epidemic threshold, we need to study the spectrum of J computed in the disease-free state ($z = x_2 = 0$):

$$J^{(\mathbf{dfs})} = \begin{pmatrix} -\mu + \lambda & 0 \\ 0 & \lambda^2 - \mu \end{pmatrix}. \tag{S.5}$$

From this we see that if $\lambda > \mu$ the *dfs* is no longer stable. Hence $T_1 = \{\lambda = \mu\}$. One could guess this without calculations from the equation in z, which tells us that the total prevalence behaves like an SIS. In order to find T_2 we now study the stability of the equilibrium where only *wt* is circulating (hosts in ⌜**wt**⌟, but not in ⌜**all**⌟, are present). This is the equilibrium **wt** defined in the main text, and it is a pure SIS model for the compartment ⌜**wt**⌟. The value of the prevalence is ($z = 1 - \mu/\lambda, x_2 = 0$), as the SIS prescribes. The Jacobian in this equilibrium point is

$$J^{(\mathbf{wt})} = \begin{pmatrix} \mu - \lambda & 0 \\ 0 & \lambda(1+\mu) - 2\mu \end{pmatrix}. \tag{S.6}$$

The first eigenvalue is always negative, as we are above T_1. The second one is negative iff $(1+\mu)\lambda < 2\mu$. As a result, we get that $T_2 = \{\lambda = 2\mu/(1+\mu)\}$.

S.2 Generic number of variants v

Assuming a generic number of variants, and homogeneous contacts, Eq. (S.1), and its Jacobian, are

$$\dot{x}_\nu = \sum_{\beta\sigma} \Lambda_{\nu\beta\sigma} x_\beta x_\sigma + \sum_\beta \Gamma_{\nu\beta} x_\beta \left(1 - \sum_\sigma x_\sigma\right) - \mu x_\nu; \tag{S.7}$$

$$J_{\nu\beta} = \frac{\partial \dot{x}_\nu}{\partial x_\beta} = \sum_\sigma \left[\Lambda_{\nu(\beta\sigma)} - \Gamma_{\nu\sigma}\right] x_\sigma + \Gamma_{\nu\beta}\left(1 - \sum_\sigma x_\sigma\right) - \mu\delta_{\nu\beta}, \tag{S.8}$$

with $\Lambda_{\nu(\beta\sigma)} = \Lambda_{\nu\beta\sigma} + \Lambda_{\nu\sigma\beta}$. They correspond to Eqs. (5) and (6) of the main text, respectively.

As in the case $v = 1$, we use the Jacobian, Eq. (S.8), to study the stability of two equilibria. The first one is the **dfs** ($x_\nu = 0$), whose analysis gives T_1. The second one is **wt**: $x_1 = 1 - \mu/\lambda$, $x_\nu = 0$ for $\nu > 1$, and will give T_2. We remind that, given the ordering we use, the index $\nu = 1$ refers to the compartment ⌜**wt**⌟, which is indeed the only one with non-zero prevalence in the *wt-phase* equilibrium. We study the stability of the former directly in the main text, so here we directly proceed to the latter.

The Jacobian computed in **wt** is

$$J^{(\mathbf{wt})}_{\nu\beta} = \left(1 - \frac{\mu}{\lambda}\right)\left(\Lambda_{\nu(\beta 1)} - \Gamma_{\nu 1}\right) + \frac{\mu}{\lambda}\Gamma_{\nu\beta} - \mu\delta_{\nu\beta}. \tag{S.9}$$

We can write it in matrix form by defining the following matrices: $(\Lambda)_{\nu\beta} = \Lambda_{\nu(\beta 1)}$, $\left(\tilde{\Gamma}\right)_{\nu\beta} = \Gamma_{\nu 1}$. The result is

$$J^{(\mathbf{wt})} = \Gamma - \mu + \left(1 - \frac{\mu}{\lambda}\right)\left(\Lambda - \Gamma - \tilde{\Gamma}\right). \tag{S.10}$$

$\tilde{\Gamma}$ has all the entries equal to $\Gamma_{11} = \lambda$ in the first row, and all others zero. This is because $\Gamma_{\nu 1}$ encodes the interactions that change the prevalence of the ν-th compartment by acting with $\ulcorner\mathbf{wt}\lrcorner$ on the susceptible state. Hence ν itself can refer only to $\ulcorner\mathbf{wt}\lrcorner$, and only wt is transmitted, thus the value λ. We now wish to show that Λ is block-upper-triangular. One diagonal block, Λ_1, encompasses the indices $\beta = 1, \ldots, 2^v - 1$, while the other, Λ_2, the remaining $\beta = 2^v, 2^v + 1$:

$$\Lambda = \left(\begin{array}{c|c} \Lambda_1\ [2^v - 1 \times 2^v - 1] & \cdots \\ \hline 0 & \Lambda_2\ [2 \times 2] \end{array}\right). \tag{S.11}$$

The lower left block is clearly zero, because the transitions that change the prevalence of $\ulcorner\mathbf{seg}\lrcorner, \ulcorner\mathbf{all}\lrcorner$ by acting on $\ulcorner\mathbf{wt}\lrcorner$ with a compartment other than $\ulcorner\mathbf{seg}\lrcorner, \ulcorner\mathbf{all}\lrcorner$, or vice versa, are not possible. The block Λ_1 is upper diagonal, and we can show this with a reasoning similar to the one for Γ in the main text. First of all, $\Lambda_{1,11} = 0$ as no term x_1^2 exists in the equations. We then consider the transitions $\ulcorner\alpha\lrcorner\ulcorner\mathbf{wt}\lrcorner \to \ulcorner\alpha\lrcorner\ulcorner\beta\lrcorner$, with $1 < \alpha, \beta \leq 2^v - 1$. It must be that $\phi(\alpha) \geq \phi(\beta)$, implying $\alpha \geq \beta$. Furthermore, the diagonal elements are $\Lambda_{1,\alpha\alpha} = \lambda^{\phi(\alpha)-1}$, as one needs to transmit all the segmented variants $\ulcorner\alpha\lrcorner$ contains, but not wt. Finally, the transitions $\ulcorner\mathbf{wt}\lrcorner\ulcorner\alpha\lrcorner \to \ulcorner\mathbf{wt}\lrcorner\ulcorner\beta\lrcorner$ are not possible, as all the compartments considered already contain the wt. This proves the upper diagonal shape. The block Λ_2 does not change in dimension with v, and so it can be computed explicitly by analyzing the four possible reactions between $\ulcorner\mathbf{seg}\lrcorner, \ulcorner\mathbf{all}\lrcorner$. Summing up, the matrices involved have the following form:

$$\Gamma = \left(\begin{array}{cccccc|cc}
\lambda & \blacksquare & \cdots & \blacksquare & \cdots & \blacksquare & \blacksquare & \blacksquare \\
0 & \lambda^2 & \cdots & \blacksquare & \cdots & \blacksquare & \blacksquare & \blacksquare \\
\vdots & \vdots & \cdots & \vdots & \cdots & \vdots & \vdots & \vdots \\
0 & 0 & \cdots & \lambda^n & \cdots & \blacksquare & \blacksquare & \blacksquare \\
\vdots & \vdots & \cdots & \vdots & \cdots & \vdots & \vdots & \vdots \\
0 & 0 & \cdots & 0 & \cdots & \lambda^v & \blacksquare & \blacksquare \\
\hline
0 & 0 & \cdots & 0 & \cdots & 0 & \lambda^v & \lambda^v(1-\lambda) \\
0 & 0 & \cdots & 0 & \cdots & 0 & 0 & \lambda^{v+1}
\end{array}\right), \tag{S.12}$$

$$\Lambda = \left(\begin{array}{cccccc|cc}
0 & \blacksquare & \cdots & \blacksquare & \cdots & \blacksquare & \blacksquare & \blacksquare \\
0 & \lambda & \cdots & \blacksquare & \cdots & \blacksquare & \blacksquare & \blacksquare \\
\vdots & \vdots & \cdots & \vdots & \cdots & \vdots & \vdots & \vdots \\
0 & 0 & \cdots & \lambda^{n-1} & \cdots & \blacksquare & \blacksquare & \blacksquare \\
\vdots & \vdots & \cdots & \vdots & \cdots & \vdots & \vdots & \vdots \\
0 & 0 & \cdots & 0 & \cdots & \lambda^{v-1} & \blacksquare & \blacksquare \\
\hline
0 & 0 & \cdots & 0 & \cdots & 0 & -\lambda & 0 \\
0 & 0 & \cdots & 0 & \cdots & 0 & \lambda(1+\lambda^{v-1}) & \lambda^v
\end{array}\right), \tag{S.13}$$

$$\tilde{\Gamma} = \left(\begin{array}{cccccc|cc} \lambda & \lambda & \cdots & \lambda & \cdots & \lambda & \lambda & \lambda \\ 0 & 0 & \cdots & 0 & \cdots & 0 & 0 & 0 \\ \vdots & \vdots & \cdots & \vdots & \cdots & \vdots & \vdots & \vdots \\ 0 & 0 & \cdots & 0 & \cdots & 0 & 0 & 0 \\ \vdots & \vdots & \cdots & \vdots & \cdots & \vdots & \vdots & \vdots \\ 0 & 0 & \cdots & 0 & \cdots & 0 & 0 & 0 \\ \hline 0 & 0 & \cdots & 0 & \cdots & 0 & 0 & 0 \\ 0 & 0 & \cdots & 0 & \cdots & 0 & 0 & 0 \end{array}\right), \tag{S.14}$$

where $2 \leq n \leq v$, and the symbol "■" marks values that are not necessary to our computation. By putting Eqs. (S.12), (S.13) and (S.14) into Eq. (S.10) we realize that $J^{(\mathbf{wt})}$ has the same block structure, and can compute its eigenvalues. The stability condition then translates into the following system:

$$\begin{cases} \mu - \lambda < 0 \\ \lambda^n - \mu + \lambda^{n-1}\left(1 - \frac{\mu}{\lambda}\right)(1-\lambda) < 0 \\ \lambda^v - \mu < 0 \\ \lambda\left(\mu\lambda^{v-1} - 1\right) < 0 \end{cases}. \tag{S.15}$$

The first equation is always true, as we are above T_1. The second one, for $n = 2$, is true when $\lambda < 2\mu/(1+\mu)$. Then, if this holds, one can show that all the following hold. As a result, we get to $T_2 = \{\lambda = 2\mu/(1+\mu)\}$.

S.3 Heterogeneous contacts

We now address the fully general equation driving the system, Eq (S.1). We assume here k to be discrete-valued. One can prove that the whole derivation holds in the continuous case, too. The general form of the Jacobian Eq. (S.1) becomes

$$\begin{aligned} J^{km}_{\nu\alpha} = \frac{\partial \dot{x}^k_\nu}{\partial x^m_\alpha} =& \frac{k}{\langle k\rangle} m p_\gamma(m) \left[\Gamma_{\nu\alpha}\left(1 - \sum_\gamma x^k_\gamma\right) + \gamma\Lambda_{\nu\alpha\gamma} x^k_\gamma\right] + \\ &+ \delta^{km}\left[-\mu\delta_{\nu\alpha} + \frac{k}{\langle k\rangle}\sum_{\beta,h} h p_\gamma(h) x^h_\beta \left(\Lambda_{\nu\beta\alpha} - \Gamma_{\nu\beta}\right)\right]. \end{aligned} \tag{S.16}$$

This matrix acts on the space $\mathcal{G} \otimes \mathcal{H}$, where $\mathcal{G}$ is the usual $(2^v + 1)$-dimensional space spanned by the compartments, and $\mathcal{H}$ is an ∞-dimensional separable Euclidean space spanned by the discrete degrees (or contact rates). For this reason, we can study the spectrum of J on the compartment sector, and the degree sector, one at the time.

Critical surface T_1

On the *dfs*, the Jacobian reads

$$J^{km}_{\nu\alpha}\Big|^{(\mathbf{wt})} = C_\gamma \frac{k m^{1-\gamma}}{\langle k\rangle}\Gamma_{\nu\alpha} - \mu\delta^{km}\delta_{\nu\alpha}. \tag{S.17}$$

In Sect. S.2 we already have examined both Γ and Λ thoroughly. Hence, we can say that the principal eigenvalue of Γ is $\lambda - \mu$, corresponding to some eigenvector v. If one defines the vector κ on $\mathcal{H}$ as simply the sequence of positive natural numbers $\kappa = (1\ 2\ 3\ \cdots)$, then one can show that $\kappa \otimes v$ is the principal eigenvector of $J|^{(\mathbf{wt})}$, with eigenvalue $\langle k^2 \rangle \lambda / \langle k \rangle - \mu$. From this we find $T_1 = \{\lambda = \hat{\mu}\}$.

Critical surface T_2

Let us call $z^k \overset{def}{=} x_1^k$. The **wt** equilibrium will be some $z^k > 0$, and $x_\nu^k = 0\ \forall \nu > 1$. This leads to (dropping the superscript **wt** from now on)

$$J_{\nu\alpha}^{km} = \frac{m p_\gamma(m)}{\langle k \rangle} k \left[\Gamma_{\nu\alpha}(1 - z^k) + \Lambda_{\nu\alpha 1} z^k \right] + \tag{S.18}$$

$$+ \delta^{km} \left[-\mu \delta_{\nu\alpha} + k \langle z \rangle_{\gamma-1} (\Lambda_{\nu 1 \alpha} - \Gamma_{\nu 1}) \right], \tag{S.19}$$

where $\langle z \rangle_\sigma$ is the average of z^k computed with $p_\sigma(k)$: $\langle z \rangle_\sigma \overset{def}{=} C_\sigma \sum_k z^k k^{-\sigma}$. By using the findings in Sect. S.2, we know that, in the compartment sector, the relevant (dominant) eigenvalue is the entry $\nu = \alpha = 2$. Hence, we can directly compute the Jacobian for these values, and deal with the degree sector:

$$J^{km} = -\mu \delta^{km} + \frac{m p_\gamma(m)}{\langle k \rangle} k \left[\lambda^2 (1 - z^k) + \lambda z^k \right]. \tag{S.20}$$

We now define two vectors (in $\mathcal{H}$): $\Omega_k \overset{def}{=} k p_\gamma(k) / \langle k \rangle$, and $\Psi_k \overset{def}{=} k \left(\lambda^2 + \lambda(1 - \lambda) z^k \right)$. With them we can rewrite J^{km}:

$$J = -\mu + \Psi \Omega^T. \tag{S.21}$$

The principal eigenvector of J is Ψ, and the corresponding eigenvalue is $-\mu + \Omega^T \Psi$. By computing it, and setting it to zero, we recover the equation for the critical point:

$$\lambda + (1 - \lambda) \langle z \rangle_{\gamma-2} = \frac{\langle k \rangle}{\langle k^2 \rangle} \frac{\mu}{\lambda}. \tag{S.22}$$

The last piece of the puzzle is computing the term $\langle z \rangle_{\gamma-2}$. We define the following function:

$$g(a, x) \overset{def}{=} \sum_{k=1}^{\infty} \frac{k^{-a}}{1 + xk}. \tag{S.23}$$

For this function, one can prove the following recursion relation:

$$x g(a - 1, x) = \zeta(a) - g(a, x) \tag{S.24}$$

(derivation not shown here), where ζ is the Riemann zeta function. Now, from [1] we know that the prevalence z^k of a SIS model obeys the following equation:

$$z^k = \frac{\lambda \langle z \rangle_{\gamma-1}}{\mu + \lambda \langle z \rangle_{\gamma-1}}. \tag{S.25}$$

We apply $C_{\gamma-1}\sum_k k^{1-\gamma}$ to both sides of this equations, and get

$$g\left(\gamma-2,\frac{\lambda}{\mu}\left\langle z\right\rangle_{\gamma-1}\right)=\frac{\mu}{\lambda C_{\gamma-1}}. \tag{S.26}$$

We then apply $C_{\gamma-2}\sum_k k^{2-\gamma}$, and get

$$\left\langle z\right\rangle_{\gamma-2}=\left\langle z\right\rangle_{\gamma-1}\frac{\lambda}{\mu}g\left(\gamma-3,\frac{\lambda}{\mu}\left\langle z\right\rangle_{\gamma-1}\right). \tag{S.27}$$

Moreover, we notice that the moments of the degree distribution can be expressed in terms of the normalization constants as follows:

$$\left\langle k^n\right\rangle=\frac{C_\gamma}{C_{\gamma-n}}. \tag{S.28}$$

By combining Eqs. (S.23), (S.26), (S.27) and (S.28), we can get to a closed-form solution for $\left\langle z\right\rangle_{\gamma-2}$:

$$\left\langle z\right\rangle_{\gamma-2}=1-\frac{\left\langle k\right\rangle}{\left\langle k^2\right\rangle}\frac{\mu}{\lambda}. \tag{S.29}$$

Finally, by putting this inside Eq. (S.22), we get to $T_2=\{\lambda=2\hat{\mu}/\left(1+\hat{\mu}\right)\}$.

S.4 Enhanced segment transmissibility

Segmented variants now transmit with a probability $\rho\lambda$, with $\rho\geq 1$, where λ is the transmissibility of *wt*. Let us examine how the interaction matrices change according to this. Matrix $\tilde{\Gamma}$ in Eq. (S.14) does not change, while matrix Γ in Eq. (S.12), and Λ in Eq. (S.13), change as follows:

$$\Gamma=\left(\begin{array}{cccccc|cc}
\lambda & \blacksquare & \cdots & \blacksquare & \cdots & \blacksquare & \blacksquare & \blacksquare \\
0 & \rho\lambda^2 & \cdots & \blacksquare & \cdots & \blacksquare & \blacksquare & \blacksquare \\
\vdots & \vdots & \cdots & \vdots & \cdots & \vdots & \vdots & \vdots \\
0 & 0 & \cdots & \rho^{n-1}\lambda^n & \cdots & \blacksquare & \blacksquare & \blacksquare \\
\vdots & \vdots & \cdots & \vdots & \cdots & \vdots & \vdots & \vdots \\
0 & 0 & \cdots & 0 & \cdots & \rho^{v-1}\lambda^v & \blacksquare & \blacksquare \\
\hline
0 & 0 & \cdots & 0 & \cdots & 0 & \left(\rho\lambda\right)^v & \left(\rho\lambda\right)^v\left(1-\lambda\right) \\
0 & 0 & \cdots & 0 & \cdots & 0 & 0 & \rho^v\lambda^{v+1}
\end{array}\right), \tag{S.30}$$

$$\Lambda=\left(\begin{array}{cccccc|cc}
0 & \blacksquare & \cdots & \blacksquare & \cdots & \blacksquare & \blacksquare & \blacksquare \\
0 & \rho\lambda & \cdots & \blacksquare & \cdots & \blacksquare & \blacksquare & \blacksquare \\
\vdots & \vdots & \cdots & \vdots & \cdots & \vdots & \vdots & \vdots \\
0 & 0 & \cdots & \left(\rho\lambda\right)^{n-1} & \cdots & \blacksquare & \blacksquare & \blacksquare \\
\vdots & \vdots & \cdots & \vdots & \cdots & \vdots & \vdots & \vdots \\
0 & 0 & \cdots & 0 & \cdots & \left(\rho\lambda\right)^{v-1} & \blacksquare & \blacksquare \\
\hline
0 & 0 & \cdots & 0 & \cdots & 0 & -\lambda & 0 \\
0 & 0 & \cdots & 0 & \cdots & 0 & \lambda+\left(\rho\lambda\right)^v & \left(\rho\lambda\right)^v
\end{array}\right), \tag{S.31}$$

The spectrum of $J^{dfs} = \Gamma - \mu$ then gives the first critical point. We find two regimes. For $\rho < \mu^{-\frac{v-1}{v}}$, we find the usual $T_1 = \{\lambda = \mu\}$. For $\rho > \mu^{-\frac{v-1}{v}}$ a different critical point emerges: $T_s = \left\{\lambda = (\mu)^{1/v}/\rho\right\}$. This means that if transmissibility is enhanced enough, the compartment ⌜**seg**⌟ spreads alone as an SIS, and T_s is its epidemic threshold.

In the regime $\rho < \mu^{-\frac{v-1}{v}}$ we can find the new $T_2 = \left\{\lambda = \frac{1+\rho}{\rho}\frac{\mu}{1+\mu}\right\}$, with the same derivation as in Sect. S.2. Analogously we can add heterogeneous contact rates, solving the degree sector as in Sect. S.3, finding the correction $\hat{\mu}$ as before.

S.5 Total prevalence of the wild-type virus

Total prevalence of the *wt* can be computed analytically in the homogeneous case. In order to prove that, we consider Eq. S.7. For convenience, we define $z = \sum_\alpha x_\alpha - x_{seg}$, which is the total prevalence of the *wt*. Primed summation symbols ($\sum'_\nu$) mean ν runs over all the compartments but ⌜**seg**⌟. We apply $\sum'_\nu$ to both sides of Eq. S.7, getting

$$\dot{z} = -\mu z + \sum_{\alpha\beta} x_\alpha x_\beta \left(\sum_\nu{}' \Lambda_{\nu\alpha\beta}\right) + (1 - z - x_{seg}) \sum_\alpha x_\alpha \left(\sum_\nu{}' \Gamma_{\nu\alpha}\right). \tag{S.32}$$

The term containing $\Lambda_{\nu\alpha\beta}$ can be computed using that $\sum_\nu \Lambda_{\nu\alpha\beta} = 0$. This is due to the fact that the number of hosts is conserved, and $\Lambda_{\nu\alpha\beta}$ encodes interactions only between infected compartments. As a result, $\sum'_\nu \Lambda_{\nu\alpha\beta} = -\Lambda_{seg,\alpha\beta}$. Moreover, one can show that $\Lambda_{seg,\alpha\beta} = -\lambda\delta_{\beta,seg}(1 - \delta_{\alpha,seg})$. The term $\sum'_\nu \Gamma_{\nu\alpha}$ is the probability of α generating a $\nu \neq seg$ by infecting a susceptible. This is just the probability of transmitting the *wt*, because all the other probabilities cancel out. Hence, $\sum'_\nu \Gamma_{\nu\alpha} = \lambda(1-\delta_{\alpha,seg})$. By inserting these two terms in Eq. (S.32), one gets

$$\dot{z} = -\mu z + \lambda(1 - z)z, \tag{S.33}$$

which decouples from the other variables, and represents a pure SIS. As a result, the endemic total prevalence of the *wt* in case of homogeneous networks is always $z = 1-\mu/\lambda$.

S.6 Numerical validation of the critical surfaces

In order to validate our theoretical prediction of the phase space, we simulate the spread of a multipartite virus on a plant population. The estimate of the critical surfaces requires computing the endemic states, corresponding to the different phases. To do that, we used the quasistationary state method [2, 3]. In its original formulation for an SIS model, the quasistationary state method relies on forcing the system out of the disease-free state. Every time the simulation produces a fully susceptible population, one inputs an active configuration previously visited by the system. With multipartite viruses, however, there is an additional challenge, represented by the fact that the disease-free state is not the only absorbing state. Every time the system becomes free of a specific variant (or *wt*) disappears from the system, it will be free of it forever. Hence, we force the system out of any state that does not contain all the v variants and the *wt*. The result of the simulations is shown in Fig. **S.1**.

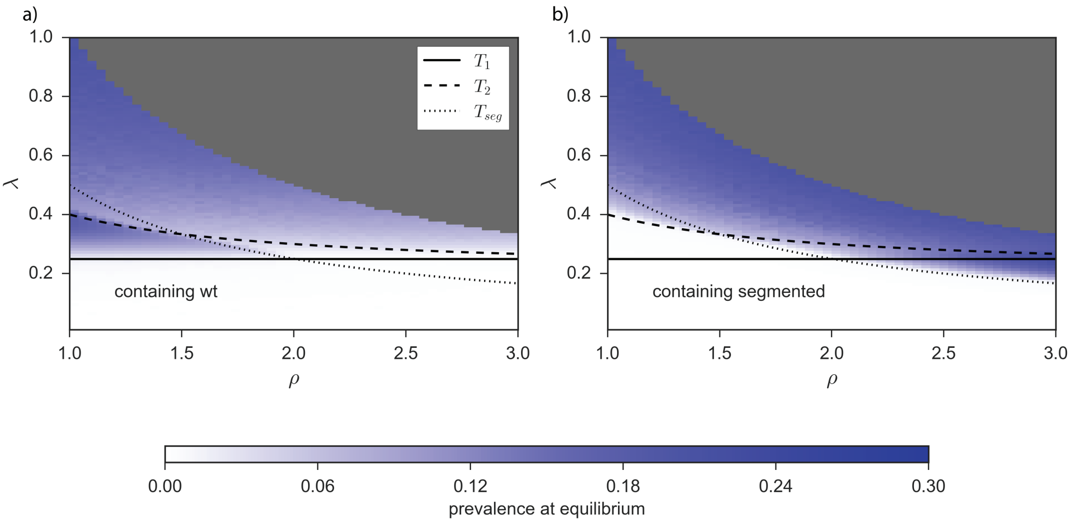

Figure S.1: Numerical validation of Fig. 3(E). Prevalence of the *wt* (A) and the segmented variants (B) are computed through stochastic simulations. The solid, dashed and dotted lines represent T_1, T_2 and T_s, respectively.

S.7 Limited carrying capacity

Our model assumes that the transmission probability of one variant does not depend on the coinfecting variants. In reality, however a limited carrying capacity should be taken into account, as the number of viral particles a cell can produce in time is limited. Here we investigate this aspect using a simple assumption: coinfecting variants share equally a fixed transmissibility. Hence, for instance, while compartment ⌜**wt**⌟ transmits the *wt* with probability λ, ⌜**1**⌟ transmits it with probability $\lambda/2$, due to the concurrent infection by a defective variant. We show that while this impacts the specific values of the critical surfaces in Eq. (2,3,4) of the main text, it does not change the quantitative behavior.

Having previously demonstrated the generalizability to arbitrary number of variants (v) and arbitrary heterogeneous topology, we set ourselves in the (computationally) simplest scenario of homogeneous network and $v = 2$. Equation (S.3) thus becomes

$$\begin{cases} \dot{z} = \lambda(1-z)z - \mu z - \frac{\lambda}{2}(1-z)y \\ \dot{x}_2 = \left(\frac{\lambda}{2}\right)^2(1-z)x_2 + \frac{\lambda}{2}(z-x_2)x_2 - \mu x_2. \end{cases} \quad . \tag{S.34}$$

From this, and from the SIS-like dynamic of ⌜**seg**⌟ spreading alone, we can compute the new critical surfaces. We consider, for simplicity, $\rho = 1$ and homogeneous topology:

$$T_1 = \{\lambda = \mu\}\,; \tag{S.35}$$

$$T_2 = \left\{\lambda = \frac{3\mu}{1+\mu/2}\right\}\,; \tag{S.36}$$

$$T_s = \left\{\lambda = v\mu^{1/v}\right\}. \tag{S.37}$$

By comparing them to Eq. (2,3,4) of the main text, we see that limited carrying capacity does not change the epidemic threshold (T_1). It increases, however, both T_2 and T_s,

making multipartitism overall less likely. It however does not change the qualitative behavior of the model.

S.8 Nonhomogeneous recovery rates, nonindependent transmission

Our model assumes recovery rate is the same for all compartment. One might instead assume that it either decreases or increases with the number of coinfecting variants. Here we investigate a different recovery rate for the pure multipartite compartment (⌜**seg**⌟): compartment containing *wt* recover at a rate μ, ⌜**seg**⌟ at a rate $\sigma\mu$.

Analogously, one might assume that variants in the pure multipartite compartment do not spread independently. To that end, we introduce another correction factor $\lambda^v \longrightarrow \alpha\lambda^v$. It is straightforward to show that both corrections impact T_2 (Eq. (4)) in the same way, with the identification $\alpha = 1/\sigma$. The new critical surface containing both factor is

$$T_s = \left\{ \lambda = \frac{1}{\rho} \left(\frac{\sigma\hat{\mu}}{\alpha} \right)^{1/v} \right\}. \tag{S.38}$$

From this we see that both these assumptions add an additional scaling to the effective recovery rate $\hat{\mu} \longrightarrow \sigma\hat{\mu}/\alpha$, while leaving the overall behavior of the model unchanged.

Supporting References

[1] Romualdo Pastor-Satorras and Alessandro Vespignani. Epidemic spreading in scale-free networks. *Phys. Rev. Lett.*, 86(14):3200–3203, 2001.

[2] Silvio C. Ferreira, Ronan S. Ferreira, Claudio Castellano, and Romualdo Pastor-Satorras. Quasistationary simulations of the contact process on quenched networks. *Phys. Rev. E*, 84:066102, Dec 2011.

[3] Silvio C Ferreira, Claudio Castellano, and Romualdo Pastor-Satorras. Epidemic thresholds of the susceptible-infected-susceptible model on networks: A comparison of numerical and theoretical results. *Phys. Rev. E*, 86(4):41125, oct 2012.
